# Medication Complexity among Disadvantaged African American Seniors in Los Angeles

**DOI:** 10.3390/pharmacy8020086

**Published:** 2020-05-16

**Authors:** Edward Adinkrah, Mohsen Bazargan, Cheryl Wisseh, Shervin Assari

**Affiliations:** 1 Department of Family Medicine, Charles R Drew University of Medicine and Science, Los Angeles, CA 90059, USA; edwardadinkrah@cdrewu.edu (E.A.); Mohsenbazargan@cdrewu.edu (M.B.); cWisseh@westcoastuniversity.edu (C.W.); 2 Department of Family Medicine, University of California Los Angeles (UCLA), Los Angeles, CA 90059, USA; 3 Department of Pharmacy Practice, West Coast University School of Pharmacy, Los Angeles, CA 90004, USA

**Keywords:** African Americans, medication complexity regimen index, older adults, black

## Abstract

**Background**. Several publications highlight data concerning multiple chronic conditions and the medication regimen complexity (MRC) used in managing these conditions as well as MRCs’ association with polypharmacy and medication non-adherence. However, there is a paucity of literature that specifically details the correlates of MRC with multimorbidity, socioeconomic, physical and mental health factors in disadvantaged (medically underserved, low income) African American (AA) seniors. **Aims**. In a local sample in South Los Angeles, we investigated correlates of MRC in African American older adults with chronic disease(s). **Methods**. This was a community-based survey in South Los Angeles with 709 African American senior participants (55 years and older). Age, gender, continuity of care, educational attainment, multimorbidity, financial constraints, marital status, and MRC (outcome) were measured. Data were analyzed using linear regression. **Results**. Higher MRC correlated with female gender, a higher number of healthcare providers, hospitalization events and multimorbidity. However, there were no associations between MRC and age, level of education, financial constraint, living arrangements or health maintenance organization (HMO) membership. **Conclusions**. Disadvantaged African Americans, particularly female older adults with multimorbidity, who also have multiple healthcare providers and medications, use the most complex medication regimens. It is imperative that MRC is reduced particularly in African American older adults with multimorbidity.

## 1. Background

Medication use is a common global practice [1]. As the world’s population is aging and the prevalence of chronic disease is increasing, medication use is also on the rise [1]. The factors that have been linked to increased medication use within the older adult population include the implementation of Medicare Part D in 2006, which provided prescription drug coverage to Medicare beneficiaries [2]; an increase in direct-to-consumer marketing of prescription drugs between 1996 and 2005, which quadrupled consumer advertising from USD 985 million to USD 4.24 billion [3], and the rise in use of cardioprotective [4] as well as antidepressant medication [5] among the elderly in the United States. An increase in medication use can lead to increased medication regimen complexity (MRC). Furthermore, it has been shown that MRC is an independent risk factor of undesired health outcomes, above and beyond other medication-related challenges such as polypharmacy, non-adherence, and inappropriate medication use [6]. 

The World Health Organization (WHO) defines medication adherence as “the extent to which a person’s behavior—taking medication, following a diet and/or executing lifestyle changes, corresponds with agreed recommendations from a healthcare provider” [6]. MRC has been identified as a risk factor for intentional non- and sometimes poor- medication adherence [7]. While inconsistencies exist about the correlation between adherence and MRC, several studies reveal that a high MRC decreases medication adherence [7,8,9]. To scientifically address the issues of complexity, the Medication Regimen Complexity Index (MRCI) was developed in 2004 to measure MRC [9]. MRCI defines MRC in three broad areas: dosage form, additional directions for medication use (e.g., need to take at a break/crush a tablet) and dosing frequency [9,10]. The MRCI enables clinicians and researchers to objectively assess prescribed and over the counter medications [11]. This index has higher validity for classifying the regimen complexity relative to a simple medication count [11,12]. This tool is also applicable in clinical research [11,12] and has been associated with chronic disease outcomes. Given the ramifications of socioeconomic status and race on increasing medical burden [13,14,15], a large proportion of low income African American (AA) older adults are required to take several complex medication regimens to manage their chronic conditions [16]. As there is a large knowledge gap regarding correlates of MRC across populations [17], there is especially a dearth of knowledge in MRC-associated factors within African American seniors. Thus, it is imperative to study the factors that affect MRC, specifically in older African American adults.

## 2. Aim

The aim of this study is to examine the sociodemographic and medical correlates that influence the medication regimen of disadvantaged African American seniors living in Los Angeles using MRCI as a measure of regimen complexity.

## 3. Methods

In this study, a survey was conducted among 709 older African American adults with chronic medical conditions to characterize the patient-level Medication Regimen Complexity Index based on prescriptions given by their providers.

### 3.1. Design and Setting

Cross-sectional data were obtained through surveys in Los Angeles between 2015 and 2018.

### 3.2. Institutional Review Board (IRB)

The study protocol was ratified by the Institutional Review Board (IRB) of the Charles R. Drew University of Medicine and Science (CDU), Los Angeles (CDU IRB #: 14-12-2450-05). Prior to enrollment, every participant endorsed a written informed consent to partake in this study. Participants received monetary compensation.

### 3.3. Process and Data Collection

Data were collected through organized face-to-face interviews and a thorough review of medications by 3 interviewers. Interviewers were assigned to participants for the purposes of data collection and follow-up during the study period. All participants were English speaking and did not require the services of an interpreter. Prior to the interview, participants were given details of the study followed by their informed consent. They were assured of anonymity and confidentiality. For the period of the interview, data on socio-demographic factors, multimorbidity, number of providers, hospitalization events and membership with a health member organization (HMO) was collected. Specifically, in assessing participants’ medication use, participants were asked to bring all over-the counter (OTC) and prescribed (Rx) medications that were taken within 2 weeks prior to the interviews. The interviewer transcribed from the container label the name of the medication, strength of the drug, expiration date, instructions, special warnings, providers’ information, etc. The medication assessment of this study employed the methodology established by Sorensen and colleagues through the drug inventory method [18]. Interviews lasted 30–70 min per session. 

### 3.4. Participants

AA seniors from South Los Angeles were recruited into this study via convenience sampling. Participants were eligible if they were 55 years or older, and could finish an English interview. Institutionalized participants and/or participants involved in any other clinical trial were omitted from the study. Participants were sampled from 16 predominantly AA churches, 11 senior housing apartment units, and (low-income) public housing projects located in Service Planning Area (SPA) 6 in Los Angeles County. Participation by community members was facilitated by religious leaders and housing residence managers. Though 740 AAs were enrolled via sampling, the present analysis was restricted to 709 AA participants who were 55 years or older.

Service Planning Area 6 was selected because up to 49% of seniors are AAs. LA County has a substantial land area (4300 sq. miles) and is therefore separated into 8 SPAs that permit the Los Angeles Department of Public Health to better carry out surveillance and deliver essential public health services that focus on the special requirements of its residents. In SPA 6, 36% and 58% of adults are uninsured and have income levels of below 200% of the federal poverty line (FPL), respectively. The prevalence of homeless AAs in SPA 6 from 2013 to 2015 has practically doubled from 39% to 70% [19].

### 3.5. Measurements

#### 3.5.1. Independent Variables

Sociodemographic Factors. Sociodemographic data captured in the study included gender, age, financial constraint, educational attainment, and marital status. Continuous variables employed were age and educational attainment (years of schooling). Greater scores suggested additional years of education. 

Financial constraint: Pearlin’s list of main chronic financial constraints was used in measuring the self-reported or perceived financial constraint experienced [20]. Questions asked included the frequency by which a participant did not have a sufficient amount of money to afford clothing or food and whether he/she had trouble paying bills. Responses went from 1 (never) to 5 (always). An additive score was computed with a greater score indicating more financial constraint. Cronbach’s alpha (reliability measured by the average covariance between item-pairs, and the variance of the total score) of the measure in this study was 0.92. 

Multimorbidity: Participants’ multimorbidity was defined by the number of chronic conditions/diseases (CD) the participant had. Multimorbidity was a total score that reflected the number of chronic conditions. Self-reports offer justifiable knowledge in regard to chronic conditions [21,22]; though, comparable to any self-reported data, a certain level of bias (under- or over-reporting) is anticipated.

Number of Providers: Our study utilized the number of providers seen as a measure for continuity of care (COC). This was based on existing literature [23] and the peculiarity of chronic disease burdens within the study population [24]. 

Hospitalization: This study variable assessed the number of times a participant was admitted in a hospital during the last 12 months.

Health Member Organization: Participants’ were asked by the interviewer of an active membership in a health member organization (HMO) insurance plan. Responses was dichotomously captured as ‘yes’ or ‘no’.

#### 3.5.2. Dependent Variables

Medication Regimen Complexity Index (MRCI): MRCI is a 65-point instrument that seeks to quantify the complexity of medications under three sections—dosage form, dosing frequency, and additional directions. The individual elements of each section are designated a numerical value/weight (range of 1.0–12.5). The weight of each variable item has been predetermined and is based on validated metrics from previous studies [9]. Each medication within the drug regimen is weighted in all 3 sections and a sum of those three sections identifies the complexity score of the said medication. The summation of individual medication complexity scores gives a total score described as the index value (MRCI) of the said set of medications/drug regimen of the participant.

### 3.6. Data Analysis

SPSS Version 23.0 (IBM Corp.: Armonk, NY, USA) was employed during data analysis. Variables were described using averages, standard deviation, and frequencies (%). We utilized Pearson correlation and independent samples *t*-test to analyze univariate correlates of MRC. First, collinearity between study variables was ruled out; then linear regression models were used for multivariable analysis. We also tested the distribution of error terms for our model. In our analysis, MRC was the dependent variable while sociodemographic (gender, age, financial constraint, marital status and educational attainment), and health-related factors (chronic conditions, hospitalization, healthcare providers) were the explanatory variables. We also reported standard errors, *t*- and *p*-values, B-regression coefficient. Standardized regression coefficient indicates how many standard deviations the outcome would change by changing one standard deviation of the independent variable. Unstandardized regression coefficient indicates how many units the outcome would change by changing 1 unit of the independent variable. Both coefficients aid in better understanding the relationship between the independent variable and outcomes.

## 4. Results

Table 1 outlines participant characteristics which comprised 709 AA seniors (aged 55–96 years) who were either male (n = 253; 35.7%) or female (n = 456; 64.3%). The participants’ average age was 72 years (SD = 8). Generally, 71.9% of them had been hospitalized within the past year. A mean of 5.9 (SD = 3.2) prescription medications (assessing polypharmacy) (1–22 range) were being taken by participants. Participants had a mean of 2.4 chronic conditions (SD = 1.3, range from 1 to 7) and visited 2.2 healthcare providers on average (SD = 1.3) annually.

Table 2 displays the findings of multivariable analysis, with multimorbidity (chronic conditions) as a categorical independent variable and the MRC as a continuous, dependent outcome. All additional variables are considered covariates. The number of prescription medications was excluded from the multivariate data analysis. The table shows that in this model, the number of chronic conditions and providers, hospitalization, and level of pain were significantly associated with higher indices of MRC after adjusting for all covariates. In addition to the number of chronic conditions, the female gender was also associated with a higher MRC, reporting 0.062 units higher in the level of MRC when compared to males (Table 2). 

As Table 3 shows, after further adjusting for demographic characteristics, hospital admission, and the number of providers, 9 out of the 13 commonly occurring chronic conditions used in this study were significantly associated with high MRC indices. 

## 5. Discussion

This study showed that in a local section of disadvantaged (medically underserved, low-income) AA seniors in South Los Angeles, higher number of chronic conditions, multiple providers, hospitalizations, and worsening levels of pain had high MRC indices. This effect was also significantly noted in gender specific MRCI correlates although advancing age, which was expected to have a bearing on MRCI showed no such relation.

Our study shows that similar to findings by Bailey et al. from a nine year follow-up, patients with multiple co-morbidities were at the greatest risk as well as frequently having complex medication regimens [25] with subsequent negative health outcomes [26]. In another study conducted among 200 community-dwelling seniors, almost half of the participants had medication regimens that were complex; the common causes identified included misunderstanding of medication instructions and polypharmacy [27]. Polypharmacy has worsened over the last three decades with prescribed medication usage doubling to four times from 1988 to 2010 [28]. The association between polypharmacy and medication regimen complexity (index) has been noted in several studies including one by Oosthuizen et al. [29]. Findings within this study further establish this significant association. The index (MRCI) thus serves as an objective proxy not just in highlighting polypharmacy but also in addressing complexities inherent in a medication prescription [30]. It fails however, to incorporate nonmedication factors such as personal [31] and social determinants of health that directly impact an older adult’s ability to fully adhere to medication [9,32].

Although underserved older African Americans are at greater risk of poor morbidity, mortality and disability outcomes, they are unlikely to be prescribed modern, more effective, and simplified combined drug regimens [33,34,35]. This paucity of access to less complicated medication regimens among disadvantaged African American seniors possibly results in the use of a greater number of older, generic medications with more complicated regimens. A review of current studies confirmed that less frequent regimens culminated in improved medication adherence and prescription profiles across pharmacotherapeutic agents [36]. 

Continuity of care (COC) is considered a foundational element of healthcare delivery especially in the primary care setting [37]. Although a small number physicians have disputed the importance of COC in this regard [38], sustained care between a provider and patient is known to improve health outcomes such as MRC, medication adherence, patient satisfaction and emergency room utilization [24,37,39,40]. Having one regular provider handle a chronic disease(s) is likely to improve a patient’s health outcome since the provider would have a relationship with the patient in addition to an in-depth grasp of the patient’s health goals [24,41]. Findings in this study point to the importance of low continuity of care by providers and MRCI patterns noted in our African American population that positively correlates the existence of a significant association. This observation is also noted in a previously conducted systematic review by Alves-Conceicao et al. [42], which identified the role healthcare providers play via the influence of MRC on health outcomes. This phenomenon is longstanding and represents a lack of or minimal communication and/or cohesion among the multiple providers who prescribe medications at different times for different reasons [43].

Studies have made conclusions like ours about high MRC in older patients, especially at the time of hospital discharge. Although there are multiple factors that account for older adult hospitalizations, the importance of addressing complexities within drug regimens is a promising avenue to curtailing risks associated with hospital readmission [1]. Based on the multiple MRCI correlates identified in this study, we concur with Tam et al. that the demand for screening and properly characterizing a complicated medication schedule in high-risk patients for follow-up medication management therapy using an objective identifier like the MRCI ultimately reduces adverse drug events (ADE) and avoidable hospital readmissions [44].

Contrary to the study from Correa-de-Araujo in 2006 [45] about the reduced likelihood of women being prescribed medications for chronic conditions as compared to men, our study had more women on complex regimens as compared to men. This difference may be explained by women having better health-seeking behavior than men [46], thus availing themselves to more providers and the likelihood of being treated for conditions that hitherto may not have been identified or treated. 

## 6. Limitations

This study obtained data based on conveniently sampled self-reports from participants who were required to recollect hospitalization events over the past year as well as corroborate chronic medical conditions listed in the survey. These may have introduced recall bias. This study utilized a cross-sectional design. Thus, no causal inference can be made between the identified sociomedical factors and MRC. Assessment of continuity of care was based solely on the number of providers. No index of measurement was utilized. Notwithstanding these limitations, the outcomes identified contributes to current literature. What we already know about MRC is limited. Finally, to avoid salami publication or duplicate publication, we did not include polypharmacy. We have previously published on polypharmacy using these data [27,47,48]. It is still an open question of by what degree polypharmacy explains medication complexity, and whether predictors of medication complexity differ between those with and without polypharmacy.

## 7. Implications and Future Research

The MRCI as a measure of MRC exists in a pool of tools being used to objectively address medically related challenges, especially in the older adult population. Our study highlights the social and medical correlates that have a bearing on the complexity and the use of such an established, valid tool. Further development of the tool is needed to address confounding variables like the personal and social determinants of health [9,32]. MRCI can be applied in seamlessly identifying potentially high-risk patients who may require targeted care and are likely to be readmitted in a hospital. Additionally, policymakers, researchers, and clinicians can utilize this tool to assess individuals at the high end of healthcare utilization patterns and poor outcomes across diverse populations.

## 8. Conclusions

From our study, disadvantaged African American seniors, particularly female older adults, participants with multimorbidity, increasing hospitalization events and impaired continuity of care, have associated high prescription-based MRC. Identification of high MRC utilizing MRCI may be a strategy to reduce morbidity and mortality due to medication-based difficulties in African American seniors with multiple chronic medical conditions.

## Figures and Tables

**Table 1:** Descriptive Characteristics (n = 709).

	**n**	**%**
**Gender**		
Female	456	64.3
Male	253	35.7
**Age**		
55–64	107	15.1
65–74	350	49.4
≥75	252	35.5
**Education**		
No high school diploma	178	25.1
High school diploma	251	35.4
Some college or college degree	280	39.5
**Living Alone**		
No	284	40.1
Yes	425	59.9
HMO Membership *		
No	459	64.7
Yes	214	35.3
**Hospitalization**		
No	199	28.1
Yes	510	71.9
		**Mean ± SD**
Age (years: 55–96)		71.9 ± 8.2
Education Attainment (1–16)		12.7 ± 2.2
Financial Constraint (1–5)		4.2 ± 1.1
Number of Providers (0–10)		2.2 ± 1.3
Number of Prescription Medications (1–22)		5.9 ± 3.2
Major Chronic Conditions (1–7)		2.4 ± 1.3
Level of Pain (0–10)		2.0 ± 2.2
Medication Regimen Complexity Index (0–45)		12.1 ± 7.3

* The number of individuals with and without health maintenance organization (HMO) membership does not equal 709—this is due to missing data.

**Table 2:** Multivariate Linear Regression Examining Association between Demographic and other Independent Variables, Excluding Number of Prescription Medications, and Medication Regimen Complexity (MRC) (N = 709).

Independent Variable	Unstandardized Coefficients		Standardized Coefficient	t Value	Sig.
	B	Std. Error	Beta		
Gender (female)	0.933	0.461	0.062	2.022	0.044
Age	0.049	0.029	0.055	1.707	0.088
Education	−0.158	0.100	−0.048	−1.581	0.114
Living Arrangement	0.534	0.455	0.036	1.174	0.241
Financial Constraint	0.125	0.233	0.019	0.534	0.593
HMO Membership	−0.226	0.462	−0.15	−0.489	0.625
Number of Providers	2.027	0.167	0.377	12.103	0.000
Hospitalization	1.360	0.522	0.083	2.602	0.009
Level of Pain	0.423	0.107	0.131	3.938	0.000
Number of Chronic Conditions	1.898	0.172	0.344	11.017	0.000
Constant	−2.488	2.610		−0.954	0.341
R Squared	0.406				

**Table 3:** Multivariate Linear Regression Examining Association between Individual Chronic Conditions and MRC, Adjusting for Demographic Characteristics, Hospital Admission, and Number of Providers (N = 709).

Chronic Conditions	Unstandardized Coefficients		Standardized Coefficient	t Value	Sig.
	B	Std. Error	Beta		
Diabetes	3.990	0.490	0.257	8.142	**0.000**
Asthma or Bronchitis	3.331	0.585	0.187	5.693	**0.000**
Heart Conditions	2.734	0.565	0.161	4.841	**0.000**
Hypertension	2.947	0.857	0.113	3.440	**0.001**
Gastrointestinal Disease	1.856	0.568	0.110	3.267	**0.001**
Stroke	2.790	0.838	0.108	3.328	**0.001**
Cancer	3.085	0.982	0.102	3.141	**0.002**
Arthritis	1.152	0.498	0.079	2.313	**0.021**
Back Pain/Injury	1.130	0.524	0.074	2.157	**0.031**
Depression	0.317	0.769	0.014	0.412	0.681
Thyroid Disorders	0.360	0.833	0.14	0.433	0.665
Insomnia	1.151	0.711	0.54	1.619	0.106
Migraine	1.436	0.836	0.057	1.718	0.086

Significant associations are shown in bold.
